# The General Composition of the Faecal Virome of Pigs Depends on Age, but Not on Feeding with a Probiotic Bacterium

**DOI:** 10.1371/journal.pone.0088888

**Published:** 2014-02-19

**Authors:** Jana Sachsenröder, Sven O. Twardziok, Matthias Scheuch, Reimar Johne

**Affiliations:** 1 Federal Institute for Risk Assessment, Berlin, Germany; 2 Institute for Molecular Biology and Bioinformatic, Charité Berlin, Berlin, Germany; 3 Friedrich-Loeffler-Institute, Greifswald - Insel Riems, Germany; Virginia Polytechnic Institute and State University, United States of America

## Abstract

**Background:**

The pig faecal virome, which comprises the community of viruses present in pig faeces, is complex and consists of pig viruses, bacteriophages, transiently passaged plant viruses and other minor virus species. Only little is known about factors influencing its general composition. Here, the effect of the probiotic bacterium *Enterococcus faecium (E. faecium*) NCIMB 10415 on the pig faecal virome composition was analysed in a pig feeding trial with sows and their piglets, which received either the probiotic bacterium or not.

**Results:**

From 8 pooled faecal samples derived from the feeding trial, DNA and RNA virus particles were prepared and subjected to process-controlled Next Generation Sequencing resulting in 390,650 sequence reads. In average, 14% of the reads showed significant sequence identities to known viruses. The percentage of detected mammalian virus sequences was highest (55–77%) in the samples of the youngest piglets and lowest (8–10%) in the samples of the sows. In contrast, the percentage of bacteriophage sequences increased from 22–44% in the youngest piglets to approximately 90% in the sows. The dominating mammalian viruses differed remarkably among 12 day-old piglets (kobuvirus), 54 day-old piglets (boca-, dependo- and pig stool-associated small circular DNA virus [PigSCV]) and the sows (PigSCV, circovirus and “circovirus-like” viruses CB-A and RW-A). In addition, the Shannon index, which reflects the diversity of sequences present in a sample, was generally higher for the sows as compared to the piglets. No consistent differences in the virome composition could be identified between the viromes of the probiotic bacterium-treated group and the control group.

**Conclusion:**

The analysis indicates that the pig faecal virome shows a high variability and that its general composition is mainly dependent on the age of the pigs. Changes caused by feeding with the probiotic bacterium *E. faecium* could not be demonstrated using the applied metagenomics method.

## Introduction

The viral community present in faeces is composed of a variety of viruses originating from the gut tissue, from intestinal microorganisms or from ingested food. The totality of viruses present in faeces has also been frequently designated as the faecal virome [1], [2]. The functions of the faecal virome are supposed to be manifold, which include roles for the viruses as pathogens, regulators of bacterial growth, gene-transfer vehicles and modulators of the immune system [3]–[6]. Early insights into the composition of the human faecal virome were provided by random cloning strategies [7], [8]. Later on, the availability of deep sequencing methods lead to more comprehensive analyses of faecal viromes [1], [9], including the development of process-controlled techniques enabling comparison of different analyses [10].

The composition of the faecal virome of pigs has been studied recently [2], [10], [11]. Although samples derived from different continents had been analysed in these studies, the general composition was found to be similar. The majority of the detected virus sequences belonged to bacteriophages and pig viruses. Only a few sequences belonged to plant viruses as well as other viruses. Most of the bacteriophage sequences originated from viruses belonging to the families *Siphoviridae*, *Microviridae* and *Myoviridae* [10]. The most abundant porcine viruses were kobuvirus, rotavirus, pig stool-associated small circular DNA virus (PigSCV), astrovirus, sapovirus and enterovirus B. Most of them represent widely distributed enteric viruses of pigs [2], [10], [11]. Whereas rotaviruses are well-known pathogens of piglets, which may lead to diarrhoea [12], [13], [14], the clinical importance of the other viruses is a subject of controversy [10], [15]–[22].

Only little is known about the stability and dynamics of the faecal virome under different conditions. For the human faecal virome, Reyes et al. [9] investigated the intra- and interpersonal variation by analysing faeces of monozygotic twins and their mothers at different time-points. By this, it was found that the viromes were unique to the individuals regardless of their degree of genetic relatedness. Minot et al. [1] analysed the inter-individual variation of the human faecal virome and its dynamic response to diet. It was shown that the largest source of variance among the viromes was caused by interpersonal variations and not by the diet. A high interpersonal diversity of gut bacteriophages was also described in two humans which were monitored over a 2.5 year period [23]. In another study, a much lower diversity of the virus community was found in infants as compared to adults [24]. Although this study has been conducted by cloning followed by classical sequencing, mathematical modelling of the derived sequence data indicated that the virome of adults was composed of approximately 2000 genotypes as compared to only 8 genotypes in one week-old infants.

The observed beneficial effects of probiotic bacteria on enteric virus infections have been recently reviewed by Colbere-Garapin et al. [6]. This includes clinical studies showing beneficial effects of probiotic bacteria in children with rotavirus-caused diarrhoea [25]–[27]. Feeding with probiotic microorganisms such as *Lactobacillus rhamnosus GG, Saccharomyces boulardii* or *Bifidobacterium lactis* resulted in milder clinical symptoms, reduced virus shedding and shortened the duration of diarrhoea in children [27]–[29]. In pigs, *Enterococcus (E.) faecium* NCIMB 10415 is a commonly used probiotic bacterium [35], [37]. It has been shown recently, that feeding of pigs with this probiotic bacterium affected shedding of enteric viruses dependent on the virus species [30]. Especially, rotavirus was shed later and in lower amounts in the group of piglets that received *E. faecium* NCIMB 10415 as compared to the control group. The specific mechanisms responsible for this effect are not known so far. However, changes in the mucosal and systemic immunity due to feeding with *E. faecium* NCIMB 10415 have been described [31]–[34]. In addition, direct interactions of this bacterium with enteric virus particles have been observed in *in vitro* studies [35]. However, it is not known so far, whether probiotic bacteria can also influence the general composition of the faecal virome, e.g. by changing the composition of the bacterial community, which represents the host population for bacteriophages, or by direct interactions with specific viruses.

The primary aim of the presented study was to analyze the effect of the probiotic bacterium *E. faecium* NCIMB 10415 on the general composition of the faecal virome in pigs. Faecal samples from sows and their piglets experimentally fed with or without the probiotic bacterium were analyzed using a process-controlled deep sequencing method. The populations of the detected virus sequences were compared between the feeding groups as well as the age groups and general insights into the stability and dynamics of the pig faecal virome under different age-related and feeding conditions were generated.

## Materials and Methods

### Ethic Statement

The animal experiment (pig feeding trial) was approved by the local state office of occupational health and technical safety “Landesamt für Gesundheit und Soziales Berlin” (LaGeSo Reg. Nr. 0347/09).

### Animal Experiment and Sampling Scheme

**Figure 1:**
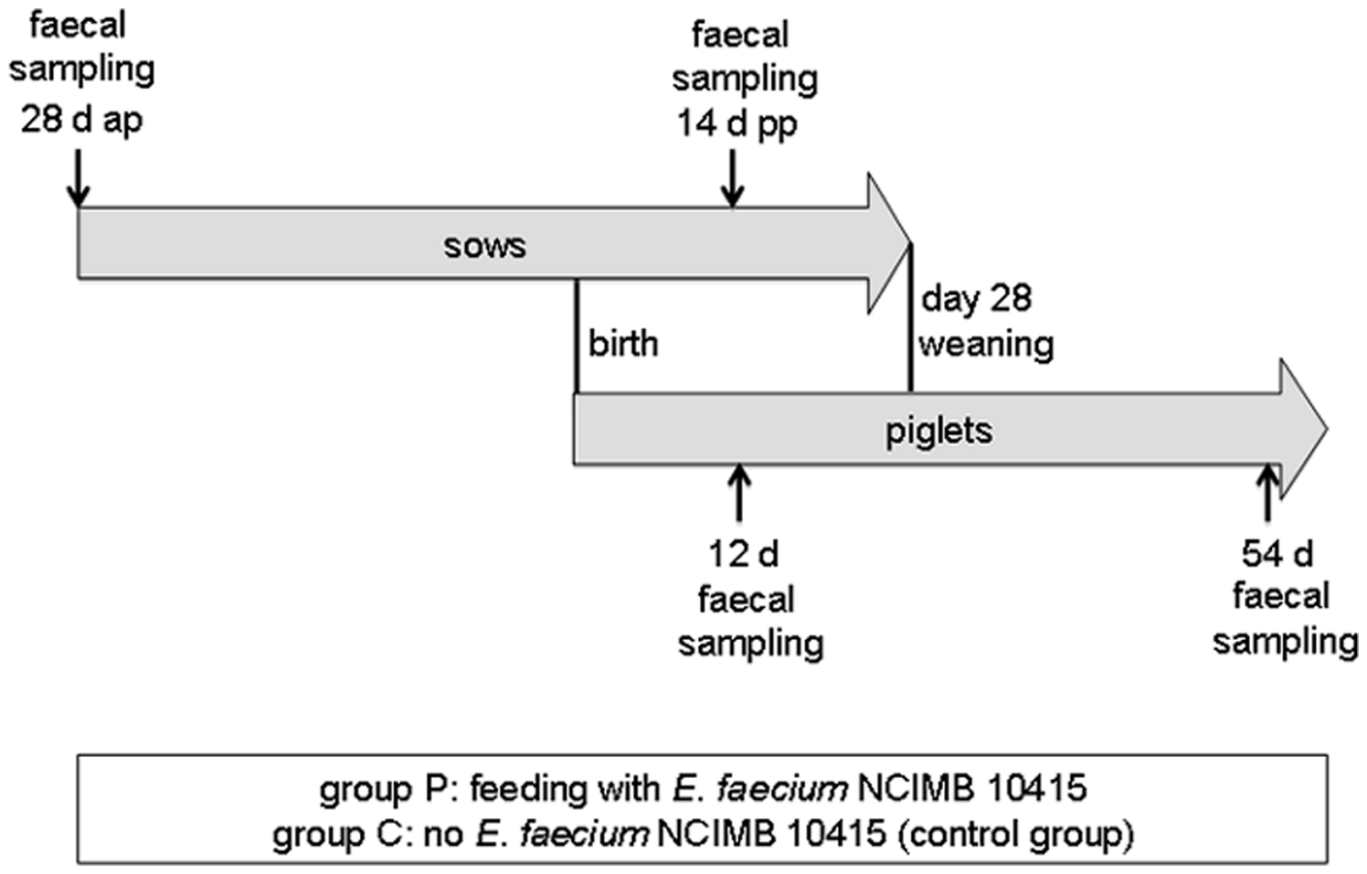
Schematic view of the experimental feeding trial. Sows and their piglets were fed with (group P) or without (group C) supplementation of the probiotic bacterium *Enterococcus faecium* NCIMB 10415. Pooled faecal samples were derived from the sows and the piglets of both groups at the indicated time-points.

The design of the pig feeding trial has been described in detail by Martin et al. [36] and is schematically shown in Figure 1. Briefly, sows and their piglets received either no probiotic bacterium or approximately 5×10^6^ cfu/g *E. faecium* NCIMB 10415, which was fed with their diet starting at 28 days ante partum. The sows received a commercial diet (UNA-HAKRA, Hamburg, Germany). Additional feeding of the piglets started at 12 days of age with a non-medicated non-commercial pre-starter diet [36]. After weaning at 28 days of age, they were fed with a non-commercial mash starter diet [37]. The homogenous distribution of the probiotic in feed has been previously demonstrated by a colony hybridization assay [37].

The faeces of 6 sows of each group were sampled at day 28 ante partum (before *E. faecium* diet) and at day 14 post partum. Faeces of their piglets (6 from each group) were collected at day 12 and at day 54 of age (end of the experiment). Piglets were euthanized at the end of the experiment by intracardial injection of a lethal dose of tetracaine hydrochloride, mebezonium iodide and embutramide (T61, Intervet, Unterschleißheim, Germany). Although the whole experiment included a larger number of animals [30], [37], only faeces of piglets were analyzed, for which the faeces of their mother sows had also been analysed. The faeces of each group and time-point were pooled. The samples were stored at −20°C until analysis.

### Quantification of *E. faecium* NCIMB 10415 in Faeces

DNA was extracted from faecal samples and subsequently analyzed by real-time PCR specific for *E. faecium* NCIMB 10415 as previously described [38]. The standards used for quantification were prepared from negative pig faecal samples spiked with known amounts of cultured *E. faecium* NCIMB 10415 cells as described by Starke et al. [37] Results are expressed as log of cell numbers per g faeces.

### Process Control

Three different bacteriophages (M13, MS2, T4) were grown, titrated and used as process controls for monitoring the efficiency of the virome analysis procedure as described previously [10]. A total of 10 µl of the bacteriophage mixture containing approximately 10^5^ plaque-forming units of each bacteriophage was added per 1 g faeces.

### Purification and Concentration of Virus Particles

Virus particles were purified from the faecal samples by a combination of tangential flow filtration (TFF) and caesium chloride (CsCl) density gradient ultracentrifugation, and concentrated by centrifugal filtration and TFF as described [10], [39]. A total of 17 g of pooled faecal samples from the sows were used. Due to limited availability of faeces in the youngest age group, 1.7 g of the pooled faecal samples from the piglets was used. The samples were spiked with test-phages and resuspended 1∶10 in SM-buffer (100 mM NaCl, 8 mM MgSO_4_, 50 mM Tris-HCl pH 7.5) by magnetic stirring. The sample was centrifuged at 10,000 g for 30 min in order to remove the large particulate debris and the supernatant was collected. The procedure was repeated by centrifugation for 3 hours at 10,000 g to remove smaller particular structures. Afterwards, a first TFF was performed using a 0.22 µm filter (PALL Corporation, Middleton; MA, USA) to remove bacterial and eukaryotic cells and debris. The remaining filtrate was subjected to a second TFF with a 50 kDa filter (PALL Corporation, Middleton; MA, USA) in order to concentrate the virus particles. The viral preparations were further concentrated by centrifugation through Vivaspin 50,000 MWCO concentrators (Sartorius Stedim Biotech GmbH, Götting, Germany) at 3,500 g resulting in a final volume of 36 ml. The preparation was divided into two fractions of 18 ml, which were added separately onto preformed stepwise caesium chloride (CsCl) density gradients with density layers of 1.7, 1.5, 1.35 and 1.2 g/ml (5 ml each) and ultracentrifuged at 20,000 g for 14 hours at 10°C. The 1.35–1.5 g/ml layers were collected from the gradients using a syringe.

### Nucleic Acid Preparation and Deep Sequencing

To eliminate free DNA present in the virus concentrate, an aliquot of 1 mL CsCl purified virus solution was treated with 50 units DNase I (2,000 U/mg, bovine pancreas grad II; Roche Diagnostics GmbH, Mannheim, Germany) for 45 min at 37°C, followed by heat inactivation for 10 min at 65°C. Thereafter, DNA and RNA were extracted simultaneously using NucliSENS magnetic extraction (bioMerieux, Nürtingen, Germany). The extracted nucleic acids (75 ng per reaction) were randomly primed for cDNA synthesis using the TransPlex® Complete Whole Transcriptom Amplification Kit (WTA2, Sigma-Aldrich, St. Louis, MO, USA) according to the protocol recommended by the supplier; however, the annealing temperature was decreased to 40°C (2 cycles) and 45°C (2 cycles) in order to enable the simultaneous amplification of DNA and RNA [10]. Aliquots of 75 µl each were removed from the WTA2 reaction at different cycle numbers, purified and size-selected using MobiSpin S-400 Columns (MoBiTec, Göttingen, Germany). The DNA concentration was measured from the preparations using a nanodrop spectrometer (Analytic Jena, Jena, Germany) and the preparation derived from a minimum of amplification cycles with a DNA concentration above 50 ng/µl was chosen for deep sequencing. A total of 1 µg DNA was used for deep sequencing on a 1/8 plate of the GS-FLX sequencer 454 Titanium (GS Titanium SV emPCR kit (Lib-L) v2; GS Titanium PicoTiterPlate Kit 70×75; GS Titanium Sequencing Kit XLR70t; Life Sciences, Roche, Branford, USA) according to the manufacturer’s protocol. The raw sequence data have been submitted to the Sequence Read Archive (SRA) at GenBank as BioProject PRJNA232620 with SRA accession numbers SRP034937 (SRX396427–SRX396434).

### Data Analysis

Primary sequence analysis was performed in two steps: identification of all virus species included in the samples and analysis of species abundances regarding selected sets of species. Raw sequence reads were subjected to primer/adaptor trimming using SeqMan (DNASTAR, Lasergene, USA) and selection for a minimum length of 50 nt. In parallel, all primary reads were subjected to de novo contig assemble using the 454 Newbler Assembler [40] software, with criteria of 90% minimum overlap identity and a minimum overlap length of 40 nt.

In order to create a local database containing all virus sequences with significant homologies to the sequence reads, homology searches for all primary reads were performed with BLASTx [41] against the non-redundant nucleotide database of NCBI [42]. In parallel, homology searches for the contigs were performed with CLC Main Workbench 6.2 [43] against the viral genome non-redundant reference sequence nucleotide database [44] and additional sequences from recently discovered viruses using the tBLASTx algorithms [39]. From both approaches, all BLAST results with an E-value < = 10^−4^ were selected and used for creation of the local sequence database.

Using this database, abundances of species were calculated. For Bray Curtis dissimilarity (see below), specific subsets, which consisted of mammalian viruses, bacteriophages or Enterococcus phages, were used. In all cases, trimmed reads were mapped against the sequences of the local database to calculate species abundances with the readmapper Bowtie 2 2.0.5 [45]. Thereafter, numbers of mapped reads were corrected for multiple read assignments. The reads of the bacteriophages used as process control were subtracted from the number of the virus reads in subsequent analyses. Shannon index [46] was calculated to compare the diversity of the species identified by primary reads. The Shannon index is maximal for a sample with a balanced species distribution and it has a low value for a sample with an uneven species distribution; e.g. if some single species are a highly abundant. The maximal value depends on the number of species in a sample. Bray Curtis dissimilarity [47] was calculated for pairwise comparisons of samples and dendrograms were constructed by hierarchical clustering with the average linkage method. This analysis included counting of detected species and determination of their taxonomy, which was also used to determine the virus hosts (bacteria, vertebrates, plants etc.).

## Results

### Detection of *E. faecium* NCIMB 10415 in Faeces

A total of 8 pooled faecal samples were derived from sows and their piglets from an experimental feeding trial with the probiotic bacterium *E. faecium* NCIMB 10415. Four of the samples were derived from animals receiving the probiotic bacterium (group P) and four samples originated from the control group that did not receive probiotics (group C). A detailed scheme of the feeding trial is presented in Figure 1.

The presence of *E. faecium* NCIMB 10415 in the faeces of sows and their piglets was analyzed by quantitative real-time PCR. As shown in Table 1, *E. faecium* NCIMB 10415 was not detected in the samples of the control group. Also, no *E. faecium* NCIMB was detectable in the sample taken from the sows of the probiotic group immediately at the beginning of the experiment (28 day ante partum) as well as in the samples from the 12 day-old piglets of this group, which were still suckled at this time-point. Considerable amounts of *E. faecium* NCIMB 10415 were demonstrated in the samples taken from the sows at 14 day post partum and from the 54 day-old piglets, both belonging to the probiotic group.

**Table 1:** Detection of *E. faecium* NCIMB 10415 in faeces of sows and their piglets.

	sows		piglets	
	28 d ap	14 d pp	12 d	54 d
group C (control)	nd^1^	nd^1^	nd^1^	nd^1^
group P (*E. faecium*)	nd^1^	6.97^2^	nd^1^	3.75^2^

1 nd = not detected.

2 quantitative real-time PCR results are expressed as decadic logarithmic numbers of cells per gram faeces according to Starke et al. [37].

d = days; ap = ante partum; pp = post partum.

### Process-controlled Deep Sequencing of Virus Genomes in Faecal Samples

**Figure 2:**
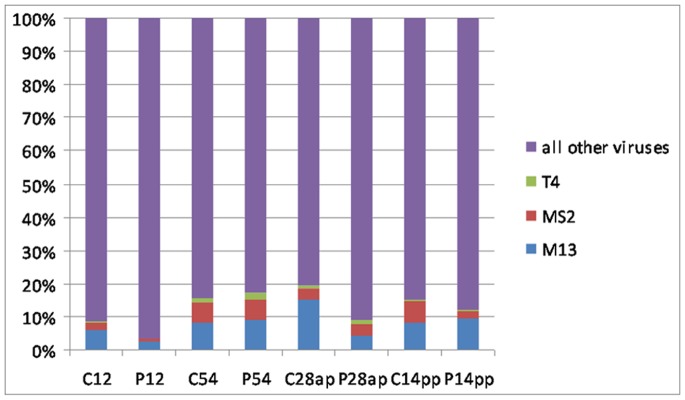
Detection of bacteriophages used as process control in the analysed samples. Equal amounts of the bacteriophages T4, MS2 and M13 were added to the pooled faecal samples prior to analysis and the generated reads were screened for the recovered genomic sequence reads of these bacteriophages. The percentage of the number of reads from these bacteriophages in relation to all detected virus reads is indicated. The samples are designated with the group letter (C – control, P – probiotic) and the day number (ap – ante partum, pp – post partum).

The 8 pooled faecal samples were analyzed by process-controlled deep sequencing. In total, 390,650 reads were generated, with an average of 48,831 reads per sample. The efficiency of the whole method was monitored by a process control consisting of three bacteriophages, which were added in constant amounts to the samples. In all samples the three test-phages could be detected representing 0.9% to 3.4% of all generated reads. The numbers of totally generated reads, test-phage reads and other virus reads is summarized for the individual samples in Table 2. The number of the test-phage reads in relation to the total virus reads ranged from 3.8% to 24.4% and is shown in Figure 2.

**Table 2:** Numbers and relative abundance of viral sequences and process control phage (test-phage) sequences in the analyzed samples.

			test-phages					without test-phages					
	samplename^1^	all reads	M13	MS2	T4	% test-phage ofall reads	% test-phage ofall viruses	viursesincl.phages	%_viusesincl.phages	viruseswithoutphages	%_viruseswithoutphages	onlybacterio-phages	%_onlybacterio-phages
piglets	C12	39670	482	168	18	1.7	9.4	7117	17.9	3981	10.0	3136	7.9
	P12	26115	221	67	11	1.1	3.8	7911	30.3	6115	23.4	1796	6.9
	C54	51534	745	552	114	2.7	18.6	7573	14.7	2387	4.6	5186	10.1
	P54	58796	1046	716	214	3.4	20.7	9535	16.2	2584	4.4	6951	11.8
sows	C28ap	64227	1085	262	63	2.2	24.4	5774	9.0	553	0.9	5221	8.1
	P28ap	55502	231	172	70	0.9	10.2	4628	8.3	429	0.8	4199	7.6
	C14pp	55851	495	378	41	1.6	18.1	5038	9.0	672	1.2	4366	7.8
	P14pp	38955	337	81	24	1.1	14.1	3143	8.1	338	0.9	2805	7.2
	average	48831	580	300	69	1.8	14.9	6340	14.2	2132	5.8	4208	8.4
	total	390650	4642	2396	555			50719		17059		33660	

1 Sample names: C–control group; P–probiotic group; 12/54 days old; 28 ap: 28 days ante partum; 14 pp: 14 days post partum.

### Analysis of Detected Virus Families and Respective Virus Hosts

**Figure 3:**
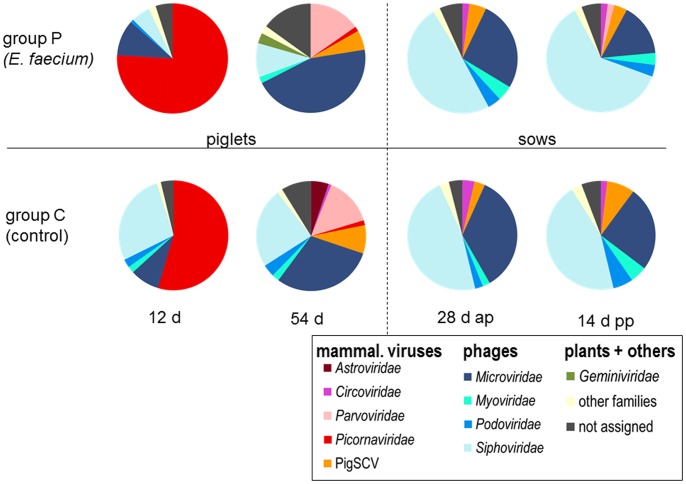
Relative abundance of virus families in the analyzed faecal viromes. The diagrams show the number of reads with sequence identities to a certain virus family in relation to all virus reads. Different colours were used for different virus families (see Legend). Virus families containing mammalian viruses are shown in shades of red, whereas those families containing bacteriophages are shown in shades of blue. The group receiving the probiotic bacterium *E. faecium* NCIMB 10415 (group P) is shown in the upper row; the control group (group C) is in the lower row. Samples derived from piglets are shown left and those from the sows are shown right. The time-points of sampling are indicated below.

Using a cut-off E-value of < = 10^−4^ for the BLASTx homology search of the sequences, the viral reads could be assigned to 36 known virus families. Only 10 of these families dominated the faecal viromes representing more than 1% in at least one of the samples. As shown in Figure 3 and Table S1, the composition of the faecal viromes according to virus families varied remarkably among the samples. A grouping of the virus families according to the taxonomic kingdom of hosts of the contained viruses revealed that the main detected groups were mammalian viruses (colored red in Fig. 3) and bacteriophages (colored blue in Fig. 3). In contrast, viruses from other hosts (insects, plants, amphibians and fungi) ranked together between 0.2% and 3.4% only.

A closer inspection of the proportion of the read numbers from mammalian viruses compared to that from bacteriophages revealed marked differences between the samples derived from different age groups. In the youngest piglet group (12 days of age), the main fraction consisted of mammalian viruses with 55% (control group) and 77% (probiotic group). In the group of 54 day-old piglets, the proportion of mammalian viruses was reduced to 24% (control group) and 30% (probiotic group). Within the four groups of the sows (one year old) the amount of mammalian viruses ranged from 8% to 12%. In contrast to those findings, the proportion of bacteriophages increased with the age of the pigs. In the 12 day-old piglets, 44% (control group) and 22% (probiotic group) of the reads relate to bacteriophages. The percentage of bacteriophages increases in the 54 day-old piglets to 68% (control group) and 72% (probiotic group), whereas approximately 90% of the virus reads belong to bacteriophages in the four sow groups. No differences in the general composition of virus families or the respective hosts were evident, when the probiotic group was compared to the control group.

### Analysis of Bacteriophages

**Figure 4:**
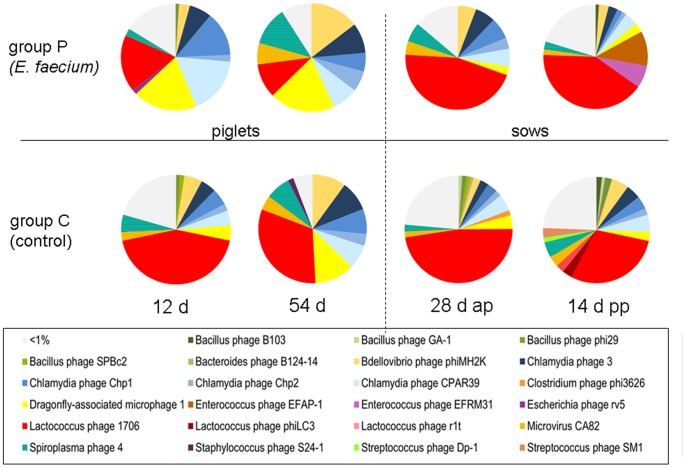
Relative abundance of bacteriophage species among all bacteriophages detected in the analyzed faecal viromes. The diagrams show the number of reads with sequence identities to a certain bacteriophage species in relation to all bacteriophage reads. Different colours were used for different bacteriophage species (see Legend). Bacteriophage species showing an abundance of less than 1% in a distinct faecal virome are subsumed in light grey colour (<1%). The group receiving the probiotic bacterium *E. faecium* NCIMB 10415 (group P) is shown in the upper row; the control group (group C) is in the lower row. Samples derived from piglets are shown left and those from the sows are shown right. The time-points of sampling are indicated below.

In overall, sequences with significant identities to 524 known bacteriophage species were detected. The bacteriophages most abundant in the eight samples are shown in Figure 4 and Table S2. In all cases, the bacteriophage population is dominated by 9 to 16 species, which represent 76–90% of all bacteriophage reads of the respective sample. The most abundant phages as identified by the highest number of reads with sequence identities to known bacteriophage genomes are Lactococcus phage 1706, Dragonfly-associated microphage 1, Chlamydia phages 4, Chp1 and Chp2, Bdellovibrio phage phiMH2K, Spiroplasma phage 4, Microvirus CA82 as well as Enterococcous phages EFAP-1 and EFRM31. A comparison between the bacteriophage populations of the specific samples indicated that many of the most abundant bacteriophage species are present in all samples, however, with different relative frequency. Apart from that, the composition of the faecal virome with regard to bacteriophage species was relatively variable between the samples and every sample contained its unique collection of bacteriophages. No consistent differences between age groups and feeding groups were obvious when the abundance of bacteriophage species was analyzed. Interestingly, the sample taken at day 14 post partum from the *Enterococcus faecium*-fed sows contained relatively high amounts of the Enterococcous phages EFAP-1 (10.8%) and EFRM31 (7.0%), which were only sporadically detected in the other groups (0.02% to 0.6%).

### Analysis of Animal Viruses

**Figure 5:**
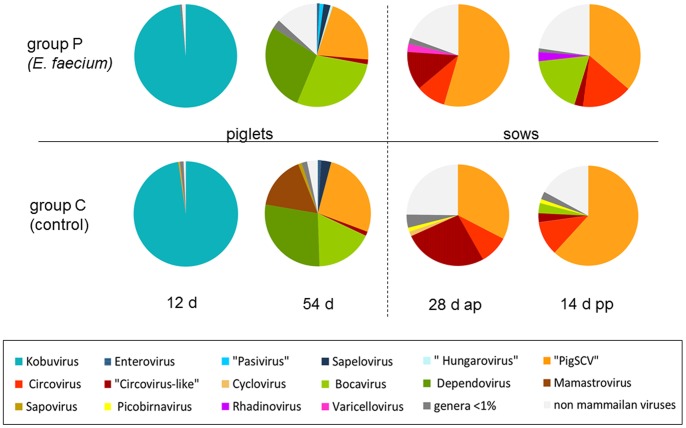
Relative abundance of mammalian virus genera among all animal viruses detected in the analyzed faecal viromes. The diagrams show the number of reads with sequence identities to a certain mammalian virus genus in relation to all animal virus reads. Different colours were used for different mammalian virus genera (see Legend). Mammalian viruses, which are so far not assigned to a certain genus, are indicated in apostrophes. Mammalian virus genera showing an abundance of less than 1% in a distinct faecal virome are subsumed in dark light grey colour (<1%). Viruses from non-mammalian hosts are subsumed in light grey colour. The group receiving the probiotic bacterium *E. faecium* NCIMB 10415 (group P) is shown in the upper row; the control group (group C) is in the lower row. Samples derived from piglets are shown left and those from the sows are shown right. The time-points of sampling are indicated below.

By analysis of all virus reads excluding the bacteriophages, sequences with significant identities to 205 known virus species were detected. Among that, 92.9% of the sequences belonged to viruses infecting mammalian animals. This percentage of mammalian viruses decreased with age, with an average of 99.1% in the 12 day-old piglet group, 91.8% in the 54 day-old piglet group and 79.0% in the sows. The relative percentage of the most abundant mammalian virus genera in the eight samples is shown in Figure 5 and Table S3, indicating that remarkable differences exist between the age groups. In the samples from 12 day-old piglets, almost all virus sequences (97.9%) belonged to porcine kobuvirus. In the samples from the 54 day-old piglets, dependovirus (27.9%), bocavirus (22.9%) and PigSCV (23.9% ) dominated the virus sequence reads. In the samples from the sows, several different small circular DNA viruses such as PigSCV (46.3%) and circovirus including “circovirus-like” viruses (22.4%) were the most abundant mammalian viruses. Among the “circovirus-like” viruses, sequences with highest identities to the viruses CB-A and RW-A were most often detected. No consistent differences were obvious between the group fed with the probiotic bacterium and the control group. However, a relatively high proportion of mamastrovirus sequences (16.3%) was detected in the sample derived from the 54 day-old piglets; while this virus was not detected in the other groups (less than 2 reads per sample).

### Analysis of Diversity and Similarity of Faecal Viromes

The calculation of the Shannon index was used to assess the diversity of the sequences detected in the specific samples (Table 3). Generally, comparison of Shannon indexes between piglets and sows indicated that the diversity increased with age. When only the bacteriophage sequences were analysed, the average Shannon index of the piglet groups was 1.3 and that of the sows 1.7. For the mammalian virus sequences, the average Shannon index for the piglets was 1.9 and that for the sows 3.0.

**Table 3:** Calculated Shannon indexes reflecting the diversity of the analyzed faecal viromes.

	Shannon index mammalia viruses		phages	
Samples^1^	minimal value	maximal value	minimal value	maximal value
C12	1,64898	3,295837	1,445446	3,091042
P12	2,158605	4,343805	1,167458	5,913503
C54	1,874457	4,634729	1,389295	6,226537
P54	2,066851	4,564348	1,300899	6,062785
C28ap	3,207631	4,812184	1,350963	6,267201
P28ap	3,127225	4,820282	1,754871	6,267201
C14pp	3,041299	4,682131	1,878738	6,23637
P14pp	2,644238	4,70048	1,732825	6,257668

1 Sample designations: C–control group; P–probiotic group; 12/54 days old (piglets); 28 ap: 28 days ante partum (sows); 14 pp: 14 days post partum (sows).

**Figure 6:**
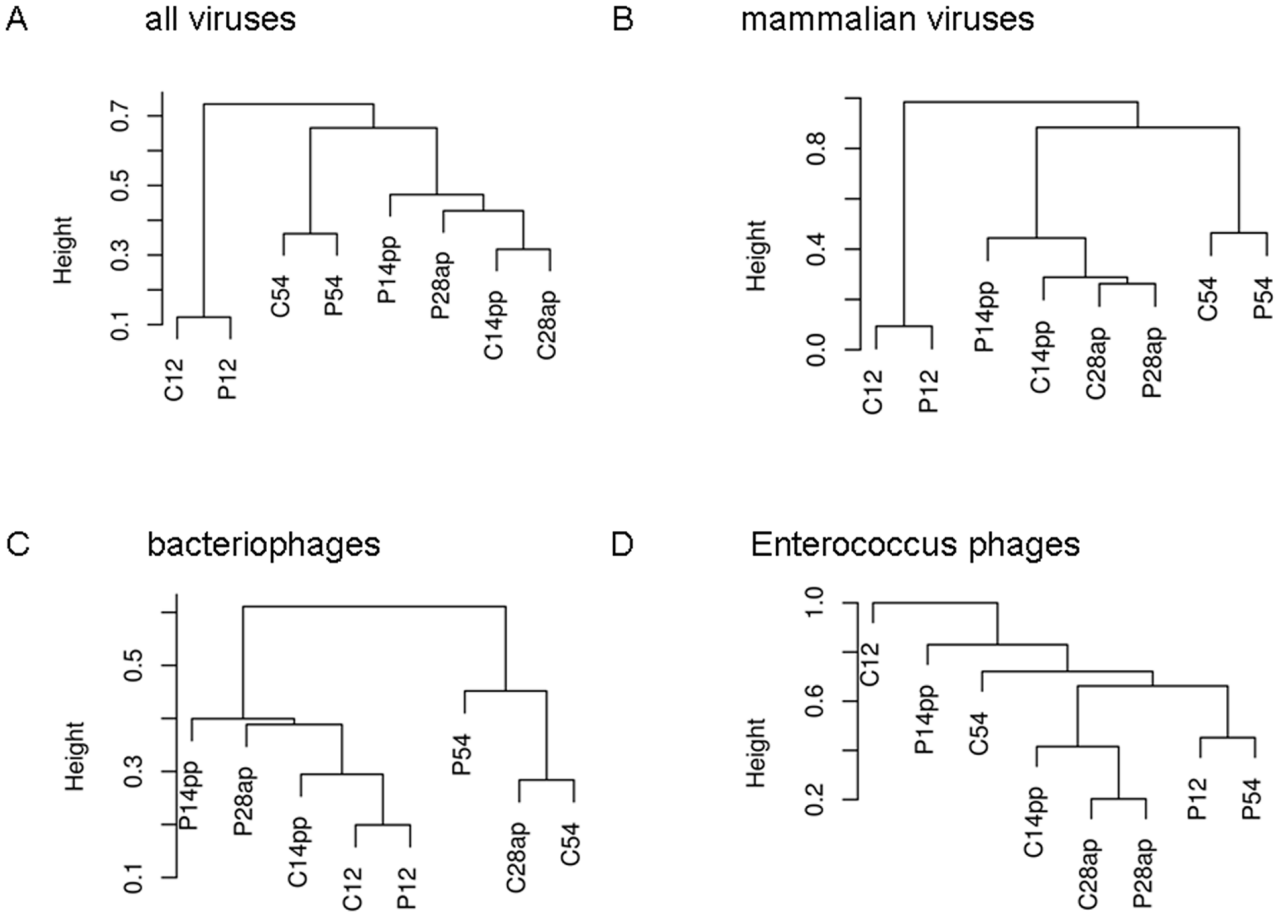
Cluster dendrograms showing the similarity of the composition of the analysed faecal viromes. The dendrograms were calculated using Bray Curtis dissimilarity. The presented dendrograms are based on a comparison of reads from all detected viruses (A), mammalian viruses (B), bacteriophages or Enterococcus phages (D). The samples are designated with the group letter (C – control, P – probiotic) and the day number (12/54 days old piglets; 28 days ante partum or 14 days post partum for sows). The dendrograms are scaled in Bray–Curtis dissimilarity units (BC; from 0 to 1).

Calculation of Bray-Curtis distances determined similarities of the faecal viromes detected in the specific samples. Figure 6 illustrates clustering of samples on the basis of Bray Curtis distance calculated by abundances of species-specific subsets. As shown in the dendrogram based on abundances of all virus species (including bacteriophages), a grouping according to age is evident (Fig. 6A). The two samples taken from the 12 day-old piglets cluster closely together and are separated from the other samples. Among these other samples, the two samples taken from the 54 day-old piglets form one separate branch, whereas the four samples of the sows are all contained in the other branch. A branching according to the feeding group is not evident from this dendrogram. The same grouping is evident, when only the mammalian virus sequences are analysed (Fig. 6B). The analysis of the bacteriophages shows no evident grouping according to age or feeding group (Fig. 6C). Also, no grouping according to age or feeding group was evident, when only the sequences of the Enterococcus phages were used for the analysis (Fig. 6D).

## Discussion

Comparisons of the composition of intestinal viromes from different samples have been only scarcely described so far. A few studies investigated individual differences of faecal viromes and the influence of diet and age in humans [1], [9], [24], whereas similar studies on faecal viromes of pigs are almost missing. Technical problems with the use of deep sequencing methods for comparative virome analyses may represent one major problem in this context [10], [48]. Here, we tried to overcome some of these problems by using a process-controlled deep sequencing approach [10]. By this, the efficiency of the analysis can be estimated for each sample, thereby enabling identification of major differences due to different performances of the method. We could show here, that all types of the bacteriophages used as process control could be detected in the final data sets of all samples. This indicates that the method has a reproducible performance and the generated data can generally be used for comparative analyses. However, the detection rates of the process control bacteriophages varied between the samples from 0.9% to 3.4%. As the detection rate of the bacteriophages is – besides technical factors - also dependent on the amount of viruses initially present in the analyzed sample, improved deep sequencing methods enabling quantitative analyses should be developed in future for comparative virome investigations.

In the eight investigated pooled samples, the overall composition of the virus community was similar to that described for other pig faecal viromes [2], [10], [11]. The two major virus groups were bacteriophages and porcine viruses, whereas plant viruses and viruses with other hosts were only rarely detected. However, large differences were detected in the ratio between bacteriophages and mammalian viruses in the distinct samples; in addition, the diversity of detected virus species varied between the analysed viromes. These data indicate that the faecal virome of pigs is not uniform and static, but shows a remarkable variability. For human faecal viromes, a high variability even between the analyzed individuals has been described [1], [9]. As only pooled faecal samples have been analyzed in the study presented here, future investigations are necessary in order to assess the inter-individual variability of faecal viromes of pigs.

The most obvious factor influencing the composition of the pig faecal virome was the age. The percentage of porcine viruses, which comprised the most abundant group in the youngest piglet samples, decreased dramatically in the samples from the older pigs. In parallel, the percentage of bacteriophages as well as the diversity of detected virus species increased by age. Interestingly, porcine kobuvirus and pig SCV, which both had been discovered only recently [10], [49], were among the most frequently detected viruses in the faecal viromes of the youngest and oldest age groups, respectively. This underlines the importance of unfocused detection systems in order to get an undistorted picture of the abundance of viruses in a sample. As all samples analyzed here originated from an experimental feeding trial, the detected virus composition may vary in comparison to field-origin samples. However, the age-specific effect was strong and very similar in both analyzed groups, which were held completely separate during the whole period of the experiment. The differences may be explained by an age-related susceptibility to specific virus infections as well as by an increasing immunity to porcine viruses due to completed virus infections with higher age. In addition, the progressive diversification of the bacterial enteric flora, which serves as the host pool for bacteriophages, would also explain the increasing diversification of the virus flora by age. An increasing diversity of the virus species in faeces of humans has already been described [24].

In contrast to the age-related effect, no clear differences could be detected in the composition of the faecal viromes according to feeding with the probiotic bacterium *E. faecium* NCIMB 10415. A relatively high percentage of Enterococcus phages in the sample derived from an *E. faecium*-feeded group may indicate multiplication of the phage due to the application of its host. This explanation may indicate that a larger amount of the probiotic bacteria may be lysed by the bacteriophages and are therefore not available for the probiotic therapy; however, this interpretation is questionable as the bacteriophages were only found in one of the samples. Also, a relative high proportion of astrovirus was found in one of the samples of the control group. Interestingly, real-time RT-PCR analyses of samples derived from the same feeding trial confirmed the presence of astrovirus exclusively in the control group [30]. However, the same study indicated later shedding of rotaviruses with lower amounts in the probiotic group as compared to the control group, which was not detected by our virome analysis. A closer inspection of the data shows that up to 10^7^ astroviruses per gram faeces were present in the samples, whereas only up to 10^5^ rotaviruses per gram were detected [30]. Therefore, a lower sensitivity of the virome analysis method may explain the discrepancies and still deeper sequencings may be necessary in future to detect more subtle changes in the faecal virome composition due to probiotic feeding.

The composition of the identified bacteriophage species in the different samples revealed no consistent pattern. However, most of the detected sequence reads showed only moderate identities to the known bacteriophage sequences present in the database. Therefore, it has to be considered that the majority of the detected sequences belong to so far unknown bacteriophages and that the identified bacteriophage species represent only their next relatives. A definitive assignment of a host to these sequences is therefore currently not possible. The quality of the database with regard to genomic sequences of bacteriophages is crucial for virome analyses. For example, the high proportion of the detected Lactococcus phage 1706 may reflect the disproportionately high abundance of those phage sequences in the database as a consequence of intensive research on these bacteriophages, which are problematic agents for the diary cheese product industry [50]. In contrast, for another highly abundant bacteriophage, the dragonfly-associated microphage, the specific bacterial host is still unknown [51]. An increase of annotated bacteriophage sequences in the databases is therefore a prerequisite for studies on the interactions between bacteriophages and their host bacteria in the gut in future. Alternatively, a deeper sequencing may enable the assembly of complete bacteriophage genome sequences from the metagenomic data set. By this, an assignment to their hosts was possible by identification of inserted host-related sequences as recently described [3].

In summary the data show a high variability of the pig faecal virome. Most obvious are age-related differences in the proportion between pig viruses and bacteriophages as well as an increasing diversification of virus species by age. Consistent differences due to probiotic feeding could not be identified by our metagenomic analysis. The results of comparative pig virome analyses may help to understand the complex interactions between viruses, bacteria and the pig within the intestinal tract. Future research should focus on the optimization of the method in order to increase its sensitivity and on the improvement of the sequence databases, especially regarding annotated bacteriophage sequences. It will be interesting to apply the optimized techniques to analyse the diversity of faecal viromes in individuals and to identify further factors like geographical origin or disease-related changes influencing its composition.

## Supporting Information

Table S1 **Relative abundance of virus families in the analyzed faecal viromes.** The table shows the number of reads with sequence identities to a certain virus family in relation to all virus reads (in %). Families showing an abundance of less than 1% in a distinct faecal virome are subsumed to other families. Families which were not classified by the International Committee on Taxonomy of Viruses (ICTV) are subsumed to not assigned. The group P received the probiotic bacterium E. faecium NCIMB 10415 (P) and the group C (C) received no probiotic.
(PDF)

Table S2 **Relative abundance of bacteriophage species among all bacteriophages detected in the analyzed faecal viromes.** The table shows the number of reads with sequence identities to a certain bacteriophage species in relation to all bacteriophage reads (in %). Bacteriophage species showing an abundance of less than 1% in a distinct faecal virome are subsumed (<1%). The group P received the probiotic bacterium E. faecium NCIMB 10415 (P) and the group C (C) received no probiotic.
(PDF)

Table S3 **Relative abundance of mammalian virus genera among all animal viruses detected in the analyzed faecal viromes.** The table shows the number of reads with sequence identities to a certain mammalian virus genus in relation to all animal virus reads (in %). Mammalian viruses, which are so far not assigned to a certain genus, are indicated in apostrophes. Mammalian virus genera showing an abundance of less than 1% in a distinct faecal virome are subsumed (genera <1%). Viruses from non-mammalian hosts are subsumed to non mammalian viruses. The group P received the probiotic bacterium E. faecium NCIMB 10415 (P) and the group C (C) received no probiotic.
(PDF)
