# Supplementary material for: The General Composition of the Faecal Virome of Pigs Depends on Age, but Not on Feeding with a Probiotic Bacterium
Source: PLoS One. 2014 Feb 19;9(2):e88888. doi: 10.1371/journal.pone.0088888 (PMC3929612; doi:10.1371/journal.pone.0088888)
Supplement: Table S1 — Relative abundance of virus families in the analyzed faecal viromes. The table shows the number of reads with sequence identities to a certain virus family in relation to all virus reads (in %). Families showing an abundance of less than 1% in a distinct faecal virome are subsumed to other families. Families which were not classified by the International Committee on Taxonomy of Viruses (ICTV) are subsumed to not assigned. The group P received the probiotic bacterium E. faecium NCIMB 10415 (P) and the group C (C) received no probiotic. (PDF) [file pone.0088888.s001.pdf]

Supplementary Table S1: Relative abundance of virus families in the analyzed faecal viromes. The table shows the number of reads with sequence identities to a certain virus family in relation to all virus reads. Families showing an abundance of less than 1% in a distinct faecal virome are subsumed to other families. Families which were not classified by the International Committee on Taxonomy of Viruses (ICTV) are subsumed to not assign. The group P received the probiotic bacterium *E. faecium* NCIMB 10415 (P) and the group C (C) received no probiotic.

	Piglets				Sows			
**Families**	**P12_%**	**C12_%**	**P54_%**	**C54_%**	**P28ap_%**	**C28ap_%**	**P14pp_%**	**C14pp_%**
Astroviridae	/	/	/	5.2	/	/	/	/
Circoviridae	/	/	/	0.8	2.0	3.6	2.0	1.9
Parvoviridae	/	/	15.3	14.4	/	/	1.9	/
Picornaviridae	75.95	54.6	1.4	1.5	/	/	/	/
SCV			5.8	8.4	5.0	3.1	3.9	8.2
Microviridae	10.5	8.7	44.9	30.1	26.7	34.95	15.7	25.2
Myoviridae	/	1.98	1.9	2.2	4.6	2.2	3.6	5.02
Podoviridae	0.95	2.4	/	3.6	3.9	2.3	3.5	5.9
Siphoviridae	5.5	27.3	10.1	23.6	49.0	47.0	61.4	44.7
Geminiviridae	/	/	3.1	/	/	/	/	/
other families	2.2	1.3	2.4	1.6	2.2	2.8	2.3	3.2
not assigned	4.9	3.8	15.1	8.9	6.7	3.95	5.8	5.8

C – control, P – probiotic
Sows: the day number (28ap – 28 days ante partum, 14pp – 14 days post partum
Piglets: day of age → 12 days old and 54 day old
