# Supplementary material for: The General Composition of the Faecal Virome of Pigs Depends on Age, but Not on Feeding with a Probiotic Bacterium
Source: PLoS One. 2014 Feb 19;9(2):e88888. doi: 10.1371/journal.pone.0088888 (PMC3929612; doi:10.1371/journal.pone.0088888)
Supplement: Table S2 — Relative abundance of bacteriophage species among all bacteriophages detected in the analyzed faecal viromes. The table shows the number of reads with sequence identities to a certain bacteriophage species in relation to all bacteriophage reads (in %). Bacteriophage species showing an abundance of less than 1% in a distinct faecal virome are subsumed (<1%). The group P received the probiotic bacterium E. faecium NCIMB 10415 (P) and the group C (C) received no probiotic. (PDF) [file pone.0088888.s002.pdf]

Supplementary Table S2 The table shows the number of reads with sequence identities to a certain bacteriophage species in relation to all bacteriophage reads. Bacteriophage species showing an abundance of less than 1% in a distinct faecal virome are subsumed (<1%). The group P received the probiotic bacterium *E. faecium* NCIMB 10415 (P) and the group C (C) received no probiotic.

	Piglets				Sows			
**Species Phagen**	**P12_%**	**C12_%**	**P54_%**	**C54_%**	**P28ap_%**	**C28ap_%**	**P14pp_%**	**C14pp_%**
<1%	16.1	23.6	9.1	16.4	20.8	13.8	20.3	24.4
Bacillus phage B103	/	/	/	/	/	/	1.03	1.6
Bacillus phage GA-1	/	1.1	/	/	/	/	/	0.98
Bacillus phage phi29	1.0	1.3	/	/	1.1	/	/	2.1
Bacillus phage SPBc2	/	1.1	/	/	1.4	/	/	/
Bacteroides phage B124-14	/	0.9	/	/	/	/	/	/
Bdellovibrio phage phiMH2K	3.2	2.2	14.6	8.9	5.3	5.8	3.1	5.3
Chlamydia phage 3	5.3	/	5.9	5.5	3.3	3.8	1.7	3.02
Chlamydia phage Chp1	13.5	3.1	5.6	6.6	4.9	6.7	1.7	3.98
Chlamydia phage Chp2	2.1	1.8	6.2	3.2	2.1	3.5	1.3	2.01
Chlamydia phage CPAR39	17.5	5.1	7.8	6.4	4.4	5.6	4.1	/
Clostridium phage phi3626	/	1.5	/	/	/	/	/	/
Dragonfly-associated microphage 1	18.98	4.1	19.5	10.5	4.5	2.6	2.99	2.8
Enterococcus phage EFAP-1	/	/	/	/	/	/	10.8	/
Enterococcus phage EFRM31	/	/	/	/	/	/	6.95	/
Escherichia phage rv5	1.6	/	/	/	/	/	/	/
Lactococcus phage 1706	17.2	47.7	10.3	28.1	43.5	45.5	40.7	29.5
Lactococcus phage phiLC3	/	/	/	/	/	/	/	3.0
Lactococcus phage r1t	/	/	/	/	/	/	/	2.5
Microvirus CA82	/	1.8	6.4	3.8	2.6	4.2	1.7	3.2
Spiroplasma phage 4	2.2	2.1	11.6	6.6	5.1	6.01	2.5	4.7
Staphylococcus phage S24-1	/	/	/	1.4	/	/	/	/
Streptococcus phage Dp-1	/	/	/	/	/	/	/	1.4
Streptococcus phage SM1	/	/	/	/	/	/	/	2.9

C – control, P – probiotic
Sows: the day number (28ap – 28 days ante partum, 14pp – 14 days post partum
Piglets: day of age → 12 days old and 54 day old
