# Supplementary material for: The General Composition of the Faecal Virome of Pigs Depends on Age, but Not on Feeding with a Probiotic Bacterium
Source: PLoS One. 2014 Feb 19;9(2):e88888. doi: 10.1371/journal.pone.0088888 (PMC3929612; doi:10.1371/journal.pone.0088888)
Supplement: Table S3 — Relative abundance of mammalian virus genera among all animal viruses detected in the analyzed faecal viromes. The table shows the number of reads with sequence identities to a certain mammalian virus genus in relation to all animal virus reads (in %). Mammalian viruses, which are so far not assigned to a certain genus, are indicated in apostrophes. Mammalian virus genera showing an abundance of less than 1% in a distinct faecal virome are subsumed (genera <1%). Viruses from non-mammalian hosts are subsumed to non mammalian viruses. The group P received the probiotic bacterium E. faecium NCIMB 10415 (P) and the group C (C) received no probiotic. (PDF) [file pone.0088888.s003.pdf]

Supplementary Table S3: Relative abundance of mammalian virus genera among all animal viruses detected in the analyzed faecal viromes. The table shows the number of reads with sequence identities to a certain mammalian virus genus in relation to all animal virus reads. Mammalian viruses, which are so far not assigned to a certain genus, are indicated in apostrophes. Mammalian virus genera showing an abundance of less than 1% in a distinct faecal virome are subsumed (genera <1%). Viruses from non-mammalian hosts are subsumed to non mammalian viruses. The group P received the probiotic bacterium *E. faecium* NCIMB 10415 (P) and the group C (C) received no probiotic.

	Piglets				Sows			
**Mammalia Virus Genus**	**P12_%**	**C12_%**	**P54_%**	**C54_%**	**P28ap_%**	**C28ap_%**	**P14pp_%**	**C14pp_%**
Kobuvirus	98.3	97.51	/	/	/	/	/	/
Enterovirus	/	/	0.8	1.04	/	/	/	/
"Pasivirus"	/	/	1.3	/	/	/	/	/
Sapelovirus	/	/	2.2	3.1	/	/	/	/
"Hungarovirus"	/	/	0.8	/	/	/	/	/
"PigSCV"	/	0.5	21.2	26.6	54.1	32.7	36.4	61.8
Circovirus	/	/	/	/	9.3	9.2	15.98	11.01
"Circovirus-like"	/	/	1.5	/	12.1	26.4	2.7	2.98
Cyclovirus	/	/	/	/	/	1.5	/	/
Bocavirus	/	/	28.3	17.5	/	/	18.5	3.3
Dependovirus	/	/	27.5	28.2	/	/	/	/
Mamastrovirus	/	/	/	16.3	/	/	/	/
Sapovirus	/	/	/	1.0	/	/	/	/
Picobirnavirus	/	/	/	/	/	1.3	/	1.3
Rhadinovirus	/	/	/	/	/	/	2.9	/
Varicellovirus	/	/	/	/	2.6	/	/	/
genera <1%	0.6	1.13	2.8	1.7	1.9	4.2	1.2	2.5
non mammailan viruses	1.2	0.7	13.2	3.3	19.3	24.8	22.8	17.1

C – control, P – probiotic
Sows: the day number (28ap – 28 days ante partum, 14pp – 14 days post partum
Piglets: day of age → 12 days old and 54 day old
